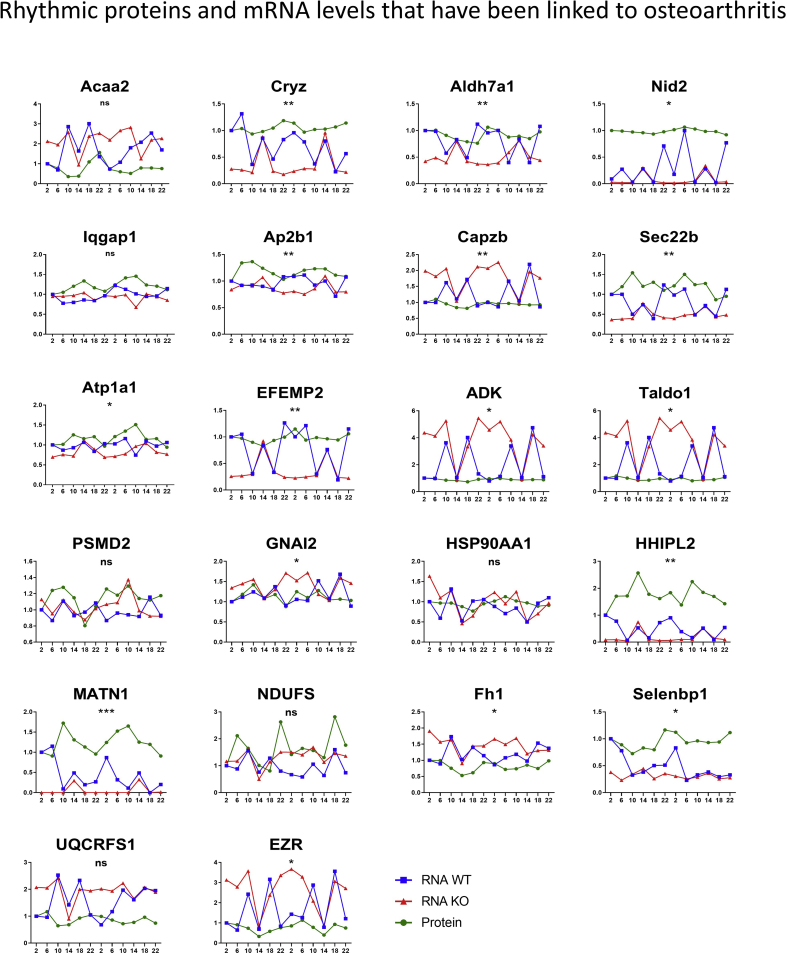# Circadian time series proteomics reveals daily dynamics in cartilage physiology

**DOI:** 10.1016/j.joca.2021.02.008

**Published:** 2021-05

**Authors:** M. Dudek, C. Angelucci, D. Pathiranage, P. Wang, V. Mallikarjun, C. Lawless, J. Swift, K.E. Kadler, R.P. Boot-Handford, J.A. Hoyland, S.R. Lamande, J.F. Bateman, Q.-J. Meng

**Affiliations:** † Wellcome Centre for Cell Matrix Research, Division of Cell Matrix Biology and Regenerative Medicine, School of Biological Sciences, Faculty of Biology, Medicine and Health, University of Manchester, Oxford Road, Manchester, UK; ‡ Division of Cell Matrix Biology and Regenerative Medicine, School of Biological Sciences, Faculty of Biology, Medicine and Health, University of Manchester, Manchester Academic Health Science Centre, Manchester, UK; § NIHR Manchester Biomedical Research Centre, Central Manchester Foundation Trust, Manchester Academic Health Science Centre, Manchester, United Kingdom; ¦ Murdoch Children's Research Institute and University of Melbourne, Parkville, Victoria, Australia

**Keywords:** Circadian clock, Cartilage, Proteomics, Osteoarthritis

## Abstract

**Objective:**

Cartilage in joints such as the hip and knee experiences repeated phases of heavy loading and low load recovery during the 24-h day/night cycle. Our previous work has shown 24 h rhythmic changes in gene expression at transcript level between night and day in wild type mouse cartilage which is lost in a circadian clock knock-out mouse model. However, it remains unknown to what extent circadian rhythms also regulate protein level gene expression in this matrix rich tissue.

**Methods:**

We investigated daily changes of protein abundance in mouse femoral head articular cartilage by performing a 48-h time-series LC-MS/MS analysis.

**Results:**

Out of the 1,177 proteins we identified across all time points, 145 proteins showed rhythmic changes in their abundance within the femoral head cartilage. Among these were molecules that have been implicated in key cartilage functions, including CTGF, MATN1, PAI-1 and PLOD1 & 2. Pathway analysis revealed that protein synthesis, cytoskeleton and glucose metabolism exhibited time-of-day dependent functions. Analysis of published cartilage proteomics datasets revealed that a significant portion of rhythmic proteins were dysregulated in osteoarthritis and/or ageing.

**Conclusions:**

Our circadian proteomics study reveals that articular cartilage is a much more dynamic tissue than previously thought, with chondrocytes driving circadian rhythms not only in gene transcription but also in protein abundance. Our results clearly call for the consideration of circadian timing mechanisms not only in cartilage biology, but also in the pathogenesis, treatment strategies and biomarker detection in osteoarthritis.

## Introduction

The night and day cycle governs the circadian (24 hourly) rhythms of rest/activity, physiology and metabolism of most animals on this planet, including humans. In mammals, a central circadian clock in the anterior hypothalamus of the brain co-ordinates the rhythmic behaviour (such as the sleep/wake cycle), and synchronizes various local circadian oscillators that are found in most peripheral organs. At the molecular level, the circadian oscillator is based on a transcriptional/translational negative feedback loop composed of interlocked transcriptional activators (BMAL1 and CLOCK) and repressors (CRYs and PERs). In addition to driving the expression of core clock genes, these clock factors control rhythmic expression of hundreds of genes in a tissue specific manner^1^. Time series transcriptome studies in mouse and primate have revealed that over 80% of protein-coding genes exhibit 24-h rhythms of mRNA expression in at least one tissue^2^.

Articular cartilage is a connective tissue at the end of long bones consisting of abundant extracellular matrix (ECM), sparsely populated by chondrocytes. Isolated from the vasculature and lacking innervations, chondrocytes are solely responsible for maintaining the fine balance between anabolic and catabolic activities within the tissue. Disruptions to this balance are implicit in the degeneration and destruction of the articular cartilage, a hallmark of joint diseases such as osteoarthritis and rheumatoid arthritis. Articular cartilages in joints are known to experience daily phases of heavy loading during activity, followed by period of low load recovery during sleep. Such daily dynamics can be manifested as rhythmic changes in cartilage thickness^3^. Interestingly, diurnal variations in cartilage metabolism has been well documented, including serum levels of COMP (cartilage oligomeric matrix protein), hyaluronic acid, keratan sulphate, aggrecan, collagen type II and TGF-β. Moreover, symptoms of arthritis, such as pain, swelling and joint stiffness have long been known to show daily rhythms^4^.

Recently, autonomous circadian clocks in chondrocytes within the articular cartilage have been demonstrated^5^^,^^6^. The cartilage circadian rhythm becomes disrupted in mouse OA models and human OA, linking circadian timing mechanism to the maintenance of homeostasis and pathogenesis of articular cartilage. Rhythmic transcriptome studies in mouse cartilage revealed a striking number of transcripts that show daily variations in their mRNA expression levels^5^. However, up until now, little is known of the scale of rhythmicity of proteins in the ECM-rich cartilage tissue. In fact, the adult articular cartilage has long been thought of as a rather static and inert tissue. It has been assumed that once the chemical structure is laid down during development, there is little change throughout the life course. Characterisation of protein dynamics during the 24-h cycle will help understand the temporal aspects of cartilage physiology and their contributions to diseases. Crucially, mass spectrometry technology has been refined to measure protein abundance in the matrix-rich cartilage tissue^7^^,^^8^. Building on these technological advances, we set out a circadian time-series proteomics analysis over multiple circadian cycles to reveal the extent of rhythmic proteins in femoral head cartilage from mice.

Our results revealed 24 h dynamics of a significant proportion of extractable cartilage proteins (12.3%), including a whole range of ECM related molecules. As a result, key physiological functions of cartilage are temporally segregated by the circadian rhythm. These findings reinforce circadian timing as a new dimension in understanding cartilage function and disease, and call for the consideration of time-of-day as a critical factor in experimental design, data interpretation, standardisation of biomarker detection and timing of therapeutic interventions in joint diseases.

## Methods

### Experimental design

In this study we harvested hip articular cartilage from 10 mice per time point every 4 h over a period of 48 h (120 samples in total). Of these, 72 samples were analysed by mass spectrometry and the rest was used for validation. The experimental design and number of biological replicates (*n* = 6 animals per time point, over 48 h) is in line with circadian time series “omics” experimental design principles as set out by the “Guidelines for Genome-Scale Analysis of Biological Rhythms”^9^. This is also in agreement with common practice in similar time series proteomics studies^10^^,^^11^. In circadian time series studies, it is well established that increasing sampling frequency and duration (two full circadian cycles) have bigger impact than increasing sample size. As stated in the Guidelines: “Although independent biological replicates increase statistical power, the high cost of “-omics” experiments can make it prohibitively expensive to collect replicate samples at each time point. Simulations indicate that replicates improve statistical power but are weaker than increasing temporal resolution if one is interested in estimating phase or amplitude”^9^.

### Preparation of cartilage samples

All animal studies were performed in accordance with the 1986 UK Home Office Animal Procedures Act. Approval was provided by the Animal Welfare Ethical Review Board (AWERB) of the University of Manchester (approval no. 50/2506). Mice were maintained at 20–22°C, with standard rodent chow available ad libitum and under 12:12 h light dark schedule (light on at 6 am; light off at 6 pm). 72 male BALB∖c mice were sacrificed at 2 months of age. Samples were taken from 6 mice per time-point every 4 h for 48 h. Articular cartilage from the head of the proximal femur was separated from the subchondral bone as described previously^5^. Cartilages from two hips of one mouse were pooled together and washed 3 times in PBS with protease (Roche 11836170001) and phosphatase inhibitors (Sigma P0044 and P5726) and subsequently snap frozen in liquid nitrogen.

### Protein extraction

Cartilage tissues were pulverized using a liquid-nitrogen-cooled tissue grinder and proteins extracted as previously described^8^. Briefly, cartilage samples were reconstituted in 100 μL of 100mM Tris acetate buffer pH 8.0 containing 10mM EDTA and protease/phosphatase inhibitors and deglycosylated by treatment with 0.1 units of chondroitinase ABC for 6 h at 37 °C. Proteins were subsequently extracted in a chaotropic buffer containing guanidine hydrochloride (4 M GuHCl, 65mM DTT, 10mM EDTA in 50mM sodium acetate, pH 5.8). Protein samples were precipitated with nine volumes of ethanol, washed once in 70% ethanol, then resuspended in 120 μL of solubilisation buffer (7M urea, 2M thiourea, and 30mM Tris, pH 8.0) and the volume was adjusted to achieve a concentration of ∼1 mg/mL, as estimated using the EZQ protein quantitation protocol (Thermo Fisher). Samples were then stored at −80 °C until required. Protein samples were analysed by SDS-PAGE and detected by silver staining as previously described.

### Peptide sample preparation and analysis by nanoliquid chromatography and LTQ-Orbitrap tandem mass spectrometry

Protein samples for LC-MS/MS analysis were sequentially reduced and alkylated under nitrogen by incubation in 10mM dithiothreitol (overnight at 4 °C) then 50mM iodoacetamide (2 h at 25 °C in the dark). Proteins were co-precipitated with 1 μg trypsin (Promega) overnight at −20 °C in 1 mL methanol. The trypsin-protein precipitates were washed once with chilled methanol, dried and reconstituted in 100mM ammonium bicarbonate, followed by trypsinization at 37 °C for 5 h, with addition of 1 μg trypsin after 2 h. Digests were terminated by freezing on dry ice. Samples were dissolved in of 0.1% formic acid, 3% acetonitrile and applied to 30 K cutoff spin filter column (Millipore Ultracel YM-30). Mass spectrometry was performed by the Mass Spectrometry and Proteomics Facility (Bio 21 Molecular Science and Biotechnology Institute, University of Melbourne). LC-MSMS was carried out on a LTQ Orbitrap Elite (Thermo Scientific) with a nanoelectrospray interface coupled to an Ultimate 3000 RSLC nanosystem (Dionex). The nanoLC system was equipped with an Acclaim Pepmap nano-trap column (Dionex – C18, 100 Å, 75 μm × 2 cm) and an Acclaim Pepmap analytical column (Dionex C18, 2 μm, 100 Å, 75 μm × 15 cm). 2 μl of the peptide mix was loaded onto the trap column at an isocratic flow of 5 μl/min of 3% CH_3_CN containing 0.1% formic acid for 5 min before the enrichment column is switched in-line with the analytical column. The eluents used for the liquid chromatography were 0.1% (v/v) formic acid (solvent A) and 100% CH_3_CN/0.1% formic acid (v/v) (solvent B). The flow following gradient was used: 6–10% B for 12 min, 10–30% B in 20 min, 30–45% B in 2 min, 45–80% in 2 min and maintained at 80% B for 3 min followed by equilibration at 3% B for 7min before the next sample injection. The LTQ Orbitrap Elite mass spectrometer was operated in the data dependent mode with nano ESI spray voltage of +2.0 kv, capillary temperature of 250°C and S-lens RF value of 60%. A data dependent mode whereby spectra were acquired first in positive mode with full scan scanning from *m*/*z* 300–1,650 in the FT mode at 240,000 resolution followed by Collision induced dissociation (CID) in the linear ion trap with ten most intense peptide ions with charge states ≥2 isolated and fragmented using normalized collision energy of 35 and activation Q of 0.25.

### Bioinformatic analysis

Maxquant version 1.5.8.3^12^ was used to analyse the raw files from the LTQ Orbitrap Elite (Thermo) with ultimate 3000 nanoLC. Spectra were searched against a Fasta file of the complete *Mus musculus* proteome downloaded from Uniprot^13^, using Maxquant internal search engine Andromeda. Settings in Maxquant for Label free quantification were left as default except that ‘match between runs’ was selected with a match time window of 2mins. Specific enzyme was Trypsin/P with max missed cleavages set to 2. Peptide and protein false discovery rates (FDR) were set to 0.01 with maximal posterior error probability (PEP) set to 0.01. The minimal peptide length was set to seven and minimum peptide and ‘razor + unique peptides’ was set to 1. Unique and razor peptides were used for quantification as recommended by Cox *et al.*^14^ with a minimum ratio count of 2. Normalised intensity values (LFQ intensity) was used for quantification and the protein groups results file was analyse using Perseus (1.5.3.1). Reverse and ‘only identified by site’ (proteins that are only identified by peptides that carry one or more modified amino acids) hits were removed. LFQ intensity data was log transformed. We only chose proteins that had MS intensity of the peptide peak available in at least 36 out of the total 72 samples for quantification. The proteomic data has been deposited to the ProteomeXchange Consortium via the PRIDE partner repository with the data set identifier PXD019431. We then use meta2d, a function of the R package *Metacycle*, to evaluate periodicity in the proteomic data. *MetaCycle* is an algorithm for detection of rhythmicity combines results of three methods that involve least-squares fits to sinusoidal curves, detecting monotonic orderings of data across ordered independent groups and autoregressive spectral estimation. The *ropls* R package^15^ was use to carry out multivariate analysis by partial least squares discriminant analysis (PLS-DA), using R2 and Q2 metrics to assess the model and significance evaluated by Y value permutation testing. Rose plots were generated in R from the proteome using metad2_*P*-value 0.05 threshold. Protein interaction network was generated in CytoScape^16^ using the STRING plugin set to high confidence cut off (0.7). Only experimentally determined interactions and from curated databases were used. Nodes without interaction partners were removed from the graph. The time of day colour coding was done after clustering based on the meta2d_phase column (Supplementary Table 1) which indicates peak of protein abundance. Considering 6 am as lights on and 6 pm as lights off for the mice. The proteins were assigned categories Morning-Early rest (phase 6.00–12), Afternoon-Late rest (Phase 12.00–18), Evening-Early active (Phase 18.00–24) and Night-Late active (Phase 0.00–6.00). ECM proteins were determined by comparison with the matrisome database (matrisome.org)^17^.

### Mouse primary chondrocyte culture

Primary chondrocytes from 5 day old mice were isolated according to published protocol^18^. Briefly, 5 day old C57bl/6 PER2:Luc mice were sacrificed by decapitation. Knee, hip and shoulder joints were dissected, and any soft tissue removed. Joint cartilage subjected to pre-digestion with collagenase D 3 mg/mL in DMEM two times for 30 min at 37°C with intermittent vortex to remove soft tissue leftovers. Subsequently, the cartilage was diced using a scalpel and digested overnight at 37°C. Cells were dispersed by pipetting and passed through 70μM cell strainer. Cell suspension was then centrifuged and the pellet was re-suspended in DMEM/F12 with 10% FBS and plated in T75 flasks. Cells were passaged only once before performing experiments.

Western blotting was performed according to standard procedures. Primary antibodies for CTGF (Abcam ab6992), PAI-1 (Abcam ab66705), MATN1 (Abcam ab106384) and alpha tubulin (Sigma T9026) were used in 1:2000 dilution. Secondary antibodies (LI-COR IRDye 800CW and 680RD) were used in 1:20,000 dilution. WB was quantified using the LI-COR Odyssey Imaging System.

## Results

**Supplementary Fig. 1:**
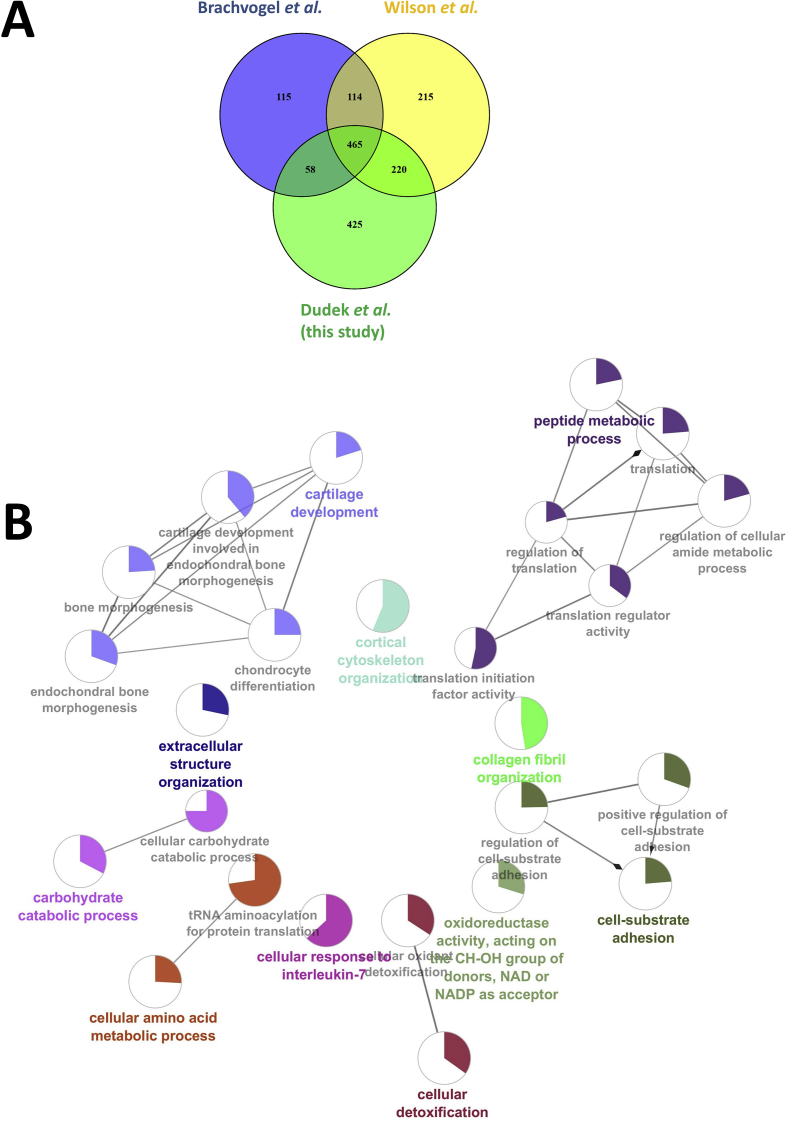
.

To identify rhythmic proteins in mouse articular cartilage, we performed a 48-h time series proteomic analysis of femoral head cartilage samples from young mice (8 weeks of age, *n* = 6 per time point) collected at 4 h intervals across two 12 h/12 h day/night cycles. Using Guanidine HCL buffer protein extraction and nanoliquid chromatography coupled tandem mass spectrometry, we were able to identify 1,177 proteins by a minimum of two unique peptides (Supplementary Table 1). 70% of identified proteins overlapped with datasets from two single time point studies using similar cartilage protein extraction methods [Supplementary Fig. 1(A)]^7^^,^^8^. GO Term analysis of the total proteome revealed significantly enriched terms including “cartilage development”, “extracellular structure organisation”, “cell-substrate adhesion”, “peptide metabolic process” [Supplementary Fig. 1(B)].

**Fig. 1:**
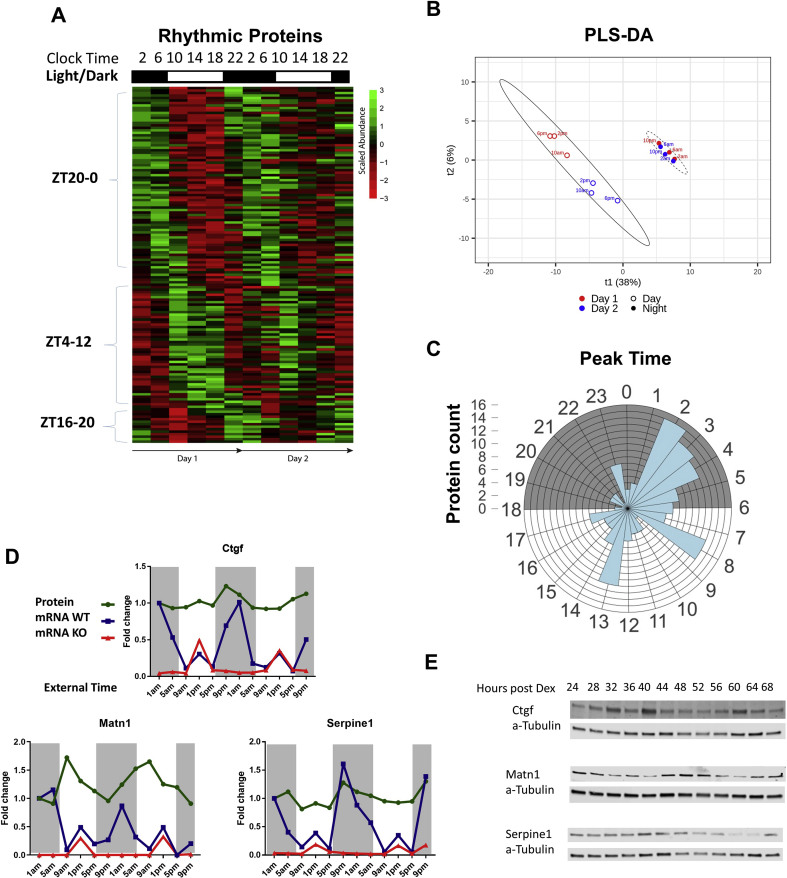
**Articular cartilage exhibits circadian rhythm in protein abundance. A.** Heat map of the 48-h time-series experiment showing 145 rhythmic proteins identified by mass spectrometry in mouse hip articular cartilage (MetaCycle integrated *P* value < 0.05). ZT – Zeitgeber Time (ZT0 = 6 am, lights on; ZT12 = 6 pm, lights off). **B.** Partial least squares discriminant analysis (PLS-DA) of the rhythmic dataset C. Rose plot showing distribution of peak abundance of rhythmic proteins within the 24-h circadian cycle. Shaded area indicates the dark phase. **D.** Fold change of protein abundance (by Mass Spec) and gene expression (by RNAseq) of selected molecules in mouse hip articular cartilage. Expression of these genes is affected by disruption of the circadian clock (Col2a1-Cre/ Bmal1 KO). Shaded area indicates the dark phase. **E.** Representative results confirming changes in protein abundance over a period of 48 h by western blotting in primary mouse chondrocyte culture synchronised by dexamethasone.

Statistical analysis of the time-series data using MetaCycle revealed 145 rhythmic proteins (12.3% of total identified) with integrated *P* value < 0.05 (Fig. 1(A) and Supplementary Table 1). Partial least squares discriminant analysis (PLS-DA) of the rhythmic dataset revealed distinct grouping of the day samples separately to the night samples and convergence of the day 1 and day two replicates [Fig. 1(B)]. Most of the rhythmic proteins peaked during late night at 2–4 am (active phase for mice, such as HSPA9, HSP90AA1, HSP90AB1, Matrilin 1, Serpine 1 and PLOD2), with notable additional peaks at 8 am (early resting phase, such as proteasome subunits PSMB7, PSMD2, PSMD5) and 1 pm (mid-resting phase, such as ribosomal proteins RPL5, RPL23a and RPS3a1) [Fig. 1(C)].

**Fig. 2:**
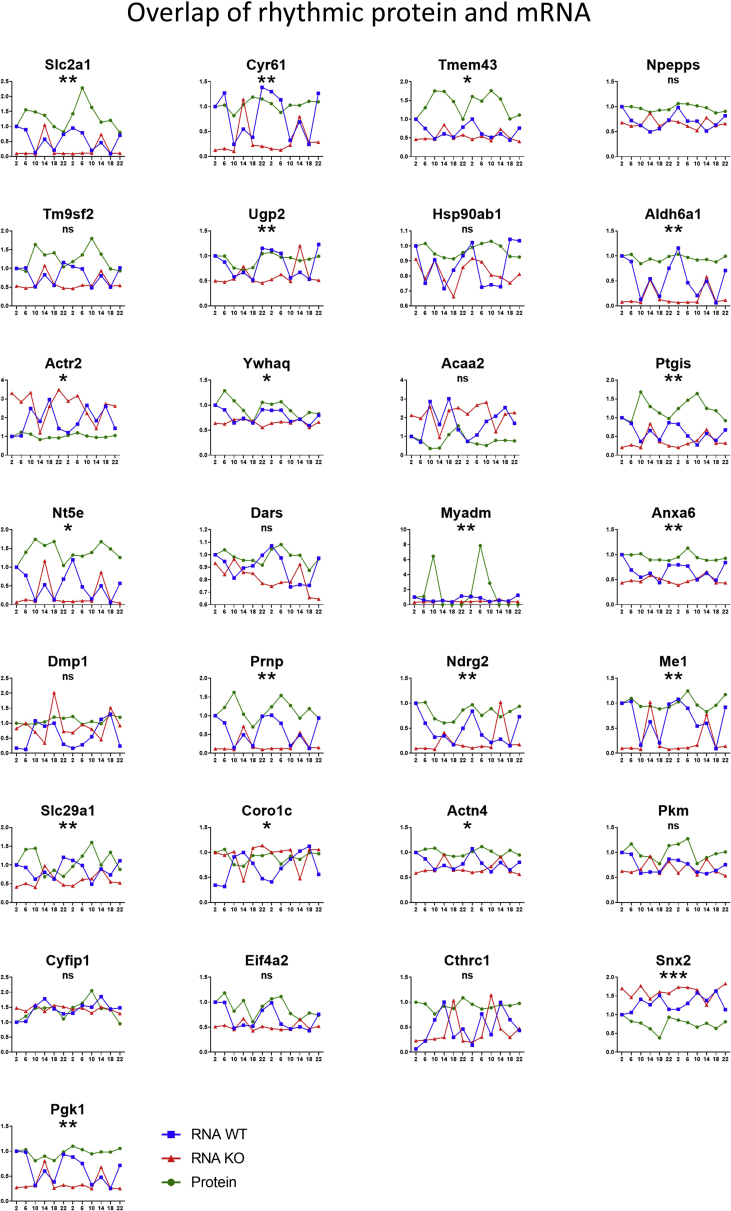
Fold change of protein abundance (by Mass Spec) and expression (by RNAseq) of genes rhythmic both on protein and mRNA level. Statistical analysis was performed to test whether there were significant differences in mRNA expression levels between the WT and Bmal1 KO mouse cartilage (∗*P* < 0.05, ∗∗*P* < 0.01, ∗∗∗*P* < 0.001, Mann–Whitney test). Please note three additional genes were presented in Fig. 1D.

Importantly, 31 of the 145 rhythmic proteins also showed rhythmicity at mRNA levels in our mouse cartilage RNAseq time-series dataset (Supplementary Tables 2 and ^5^), including CTGF, Matrilin 1 and Serpine 1. The patterns of protein and mRNA changes for CTGF and Serpine 1 mostly overlap [Fig. 1(D)]. While for Matrilin 1, a phase delay in protein peaks relative to mRNA was observed [Fig. 1(D)]. The mRNA expression levels for all three proteins were reduced in the cartilage specific *Bmal1* KO (cartilage-clockless, but behaviourally still rhythmic) mouse and their rhythmic patterns were lost [Fig. 1(D)]. In addition, daily variations in protein abundance of these molecules were independently validated in clock-synchronized mouse primary chondrocytes by western blotting [Fig. 1(E)], indicating that such clock regulatory mechanisms are cell intrinsic. Therefore, the rhythmicity of these molecules is not merely a result of passively responding to daily mechanical loading, but instead being regulated by the endogenous cartilage clock. Overall, 21 of the 31 overlapping rhythmic genes had significantly different mRNA expression in the *Bmal1* KO cartilage (*P* < 0.05, Mann–Whitney test) (Fig. 2) suggesting that at least this subset of genes is under direct control of the molecular circadian clock.

**Fig. 3:**
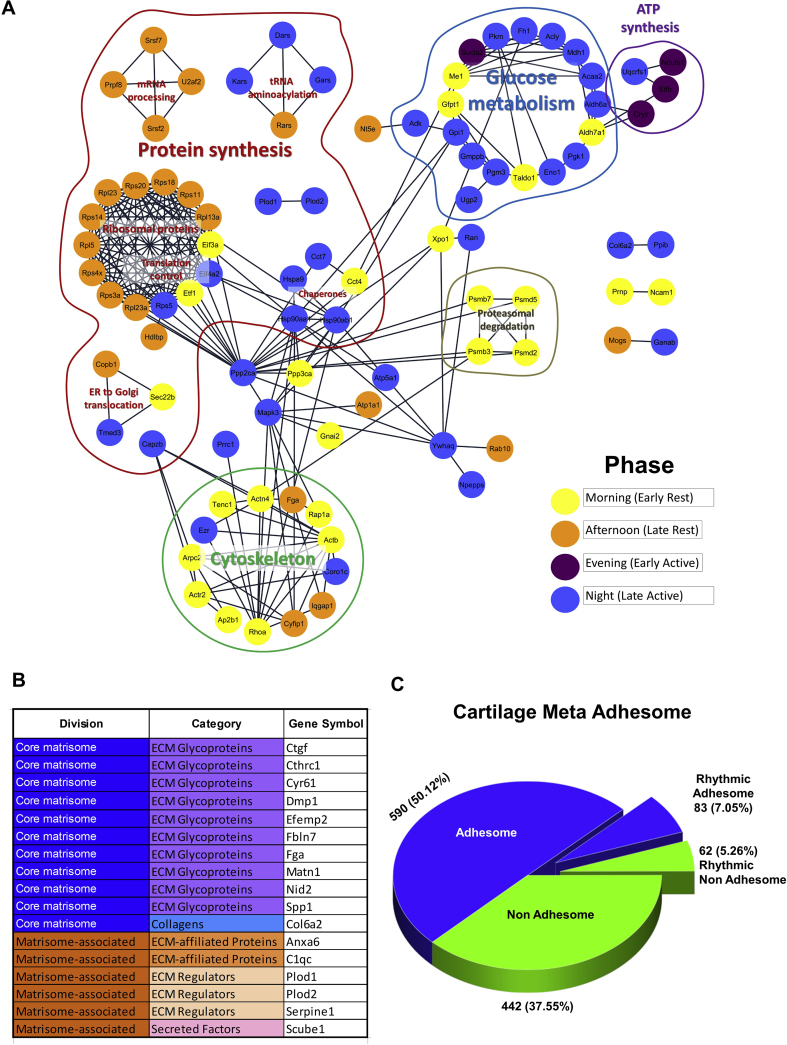
**Protein interaction network of rhythmic proteins shows time of day partitioning of cellular processes. A.** Protein interaction network generated from the 145 rhythmic proteins using the STRING plugin for CytoScape utilising interactions from experimental data and curated databases. Nodes are colour coded by peak time of protein abundance. **B.** List of rhythmic extracellular matrix proteins identified as core matrisome or matrisome-associated components. **C.** Pie chart showing proportion of rhythmic adhesion associated proteins as compared with the meta-adhesome.

STRING interaction analysis of the rhythmic proteins indicate that many proteins that physically interact with each other and take part in the same biological processes largely peak together during the same part of the day [Fig. 3(A)]. Proteins responsible for mRNA processing and ribosomal proteins peak in the afternoon. Proteins related to ATP synthesis peak in the evening (early active phase) and glucose metabolism peaks late at night (late active phase). Proteins associated with the cytoskeleton peak in the morning (early resting phase). These data reveal a temporal segregation of different metabolic events in mouse articular cartilage within the 24-h day. Analysis of the STRING interaction network revealed PPP2CA as a protein with the highest number of interaction partners (22 partners, network average 5.17), positioning as a possible signalling hub. Besides PPP2CA, the top 15 interactors were ribosomal proteins and translation factors highly interlinked with each other, followed by HSP90 (12 interactors), ACTB and RHOA (both 10 interactors).

Articular cartilage is a connective tissue with a relatively small proportion of chondrocyte cells embedded in a vast pool of ECM. Although many ECM proteins were thought to be relatively stable, when the rhythmic proteomics dataset was compared with the matrisome database (http://matrisome.org/)^19^, we were able to detect a number of ECM-related molecules that show clear daily changes in their abundance [Fig. 3(B)], including CTGF, CYR61 (CCN1), DMP1, Osteopontin, PLOD1 and PLOD2 which play key regulatory functions in cartilage. For instance, PLOD1 and 2 are responsible for hydroxylation of lysine during collagen synthesis, supporting a role of circadian rhythm in regulating the post-translational modification of ECM components. We also compared our data with consensus adhesome proteins (molecules common between proteomic datasets generated in independent studies analysing the integrin adhesion complex)^20^. Out of a list of 60 consensus adhesome molecules, five turned out rhythmic (ACTN4, HP1BP3, IQGAP1, PPIB and RPL23A). Interestingly, when compared with a list of the wider meta-adhesome (list of all molecules identified in integrin adhesion complex analysis in multiple independent studies)^20^, 83 out of 145 of our rhythmic cartilage proteins overlapped with the meta-adhesome, suggesting that a large portion of the cartilage rhythmic proteome may play a role in the dynamics of chondrocyte-ECM interactions [Fig. 3(C) and Supplementary Table 3].

Although the size of the rhythmic protein dataset precludes appropriate GO Term analysis, we looked for similarities of the processes in which the proteins were grouped in the STRING analysis with overrepresented GO Terms in the rhythmic mRNA dataset (Supplementary Table 4). Most notable among these were the “cell adhesion” and “response to unfolded protein” GO terms which supports the notion that the circadian clock may prepare chondrocytes to periods of mechanical loading and increased demands in protein synthesis. Moreover, among the 31 genes that are rhythmic both at protein and mRNA levels Actn4, Coro1c, Cyr61, Dmp 1, Myadm and Serpine1 belong to the term “cell-substrate adhesion”. Another overrepresented GO Term in the mRNA dataset was “response to unfolded protein”, which overlaps with the large portion of the STRING network dedicated to protein synthesis, including molecular chaperones. Rhythmic coordination of global protein synthesis has been described before in mouse tendon and liver, as well as in yeast^21^, ^22^, ^23^. Other aspects of metabolism may also be under the control of the circadian clock. Namely, Acaa2, Pgk1, Npepps and Ptgis belong to the term “cellular response to hypoxia”, raising the possibility that chondrocytes may go through phases of hypoxia throughout the 24 h day/night cycle and the circadian clock may help anticipate this. Indeed, rhythmic tissue oxygenation has been reported in other tissues^24^. In addition, Slc2a1 (GLUT1) and the glycolytic enzyme pyruvate kinase (PKM), both rhythmic at the protein and mRNA levels, are of pivotal importance for the switch from the highly efficient oxygen-dependent oxidative phosphorylation to nonoxygen-dependent anaerobic energy generation^25^.

Previously, it has been shown that circadian rhythms are disrupted during ageing or in osteoarthritic joints^5^. To reveal the functional relevance of the rhythmic proteins we identified in cartilage in a disease setting, we compared our rhythmic proteins with published ageing or osteoarthritis-regulated cartilage proteomic studies. This comparison revealed 30 rhythmic proteins being dysregulated in a disease condition and/or in aging (Supplementary Table 5). Four of the proteins (FGA, ANXA6, ATP1A1 and ALDH7A1) were significantly dysregulated in more than one proteomics study (highlighted red). 23 of the 30 rhythmic proteins had significantly different (*P* < 0.05, Mann–Whitney test) mRNA levels in the Bmal1 KO mouse cartilage compared to the WT, although only 9 were actually significantly rhythmic in the WT (Fig. 2 and Supplementary Fig. 2), suggesting an indirect or non-rhythmic role of the circadian factor BMAL1 in regulating their expression. These results underline the functional importance of the rhythmic cartilage molecules in cartilage physiology and disease.

## Discussion

To the best of our knowledge, our study represents the first to characterise diurnal proteome of the articular cartilage. Circadian proteomic studies have been performed in other tissues (e.g., liver, SCN, heart, plasma) which revealed profound dynamics in protein abundance and phosphorylation. Around 6% of proteins in the liver, 7.8% in the heart muscle and 11% in the SCN were found to show 24-h rhythmic patterns. In the liver, in addition to tissue specific pathways, proteins involved in ribosome biogenesis, protein folding and vesicle trafficking were found to be rhythmic, suggesting time of day dependence of protein synthesis and secretion^23^^,^^26^.

The discovery that 12% of all extractable cartilage proteins from mouse femoral head show a daily 24-h rhythm indicate that the articular cartilage is a much more dynamic tissue than previously thought, with many proteins being synthesized and/or turned over on a daily basis. This is in line with a recent study by Chang J *et al.*^21^ showing that ∼10% of tendon proteome is rhythmic and collagen type I is rhythmically secreted and assembled. Out of 145 rhythmic proteins, 31 showed rhythmicity at the transcript level in our previously published RNAseq dataset. This limited overlap between rhythmic transcripts and proteins is not surprising as several studies comparing rhythmic transcriptome and proteome have shown this disparity^27^. Daily changes in protein abundance may be a result of circadian regulation at multiple stages. Recent studies show that mammalian cells exhibit rhythmicity in processes such as mRNA transcription^28^, RNA splicing^29^, mRNA polyadenylation and stability^30^, as well as translation^31^, secretion^21^, autophagy^32^ and proteasomal degradation^33^. Additionally, nature of the cartilage tissue (rich in matrix, scarce in cells) dictates that peptides from the highly abundant ECM proteins outnumber less abundant intracellular proteins and possibly mask some of the rhythmic changes. An unbiased approach to quantifying nascent proteins could provide a more complete picture of rhythmic synthesis of more stable and accumulated proteins.

The loss of rhythmicity of mRNA levels for *Ctgf*, *Matrilin 1* and *Pai 1* in the cartilage-*Bmal1* KO mice support an essential role of the molecular clock gene in regulating rhythmicity. Further, we showed that rhythmic changes in protein abundance of these three molecules were maintained for at least 68 h in culture of clock synchronised mouse primary chondrocytes devoid of any additional external influence, such as mechanical loading. This lend additional support that levels of CTGF, Matrilin 1 and Pai 1 are indeed under the control of the endogenous chondrocyte circadian clock and not merely reacting to external factors. Interestingly, CTGF was shown to have a short half-life of 60–90 min^34^ supporting the idea that these proteins may be rhythmically synthesised and turned over.

mRNA expression of *Cyr61* and *Ctgf* have been shown to be mechanically regulated in *ex vivo* explant tissues or in cells^35^, and in human muscle *in vivo* after a single bout of exercise^36^. As such, it raises the possibility of potential involvement of diurnal loading pattern (which itself is controlled by circadian rhythm) in regulating rhythmic protein levels in cartilage. When we plotted the mRNA expression patterns of all rhythmic proteins with overlapping rhythmic mRNA, we saw an overwhelming majority of genes showing peak of expression occurring during the night timepoints (10 pm, 2 am and 6 am, the active phase for mice). However, their expression was low at these time points in the *Bmal1* KO mouse (Fig. 2). Of note is that the behavioural rhythms in the conditional knockout mice are unaffected outside the *Col2a1* expressing tissues. As such, these KO mice retain rhythmic activity (in wheel-running) just like the WT mice^5^. Therefore, at least in the portion of the dataset that shows overlapping rhythmicity at mRNA and protein levels, their rhythmic patterns are more likely to be controlled by the circadian clock and not merely by mechanical loading. However, we cannot rule out the possibility that other proteins in the dataset are rhythmic due to mechanical loading. Future proteomic studies could compare the daily gene expression patterns in mechanically loaded and unloaded mice^37^, or alternatively in wild-type and circadian clock knock-out mice.

In our cartilage proteomics analysis, we showed that a large portion of the protein synthesis machinery exhibited a circadian pattern in abundance. Most notably 11 ribosomal proteins were found to be rhythmic, of which all but one peaked at the same time. This is in line with previous reports that mouse liver exhibits daily cycles in ribosome assembly accompanied by diurnal changes in rRNA and ribosomal protein abundance, leading to daily changes in the global rate of protein translation^23^^,^^38^. Most of the rhythmic cartilage proteins peak during late active phase which indicates possible adaption of chondrocyte function to the 24 h day. Such an adaptation may allow animals to anticipate and respond to bouts of activity and related demand for protein synthesis and tissue repair thereafter. Interestingly, several rate limiting enzymes in the glycolysis pathway exhibit rhythmicity. For example, GPI catalyses the second step in glycolysis and PGK catalyses one of the two ATP producing reactions in the glycolytic pathway^39^. The ratio between the highly active tetrameric form and nearly inactive dimeric form of PKM determines whether glucose carbons are channelled to biosynthetic processes or used for glycolytic ATP production^39^. The main transmembrane glucose transporter GLUT1 (Slc2a1) was also rhythmic in our dataset and peaked roughly at the same time as the glycolytic enzymes. Considering the fact that glycolysis serves as the main source of ATP for chondrocytes^40^, circadian rhythmicity both in transport and glycolytic enzymes may have significant effects on cartilage metabolism^25^. Analysis of the STRING interaction network revealed PPP2CA as the most interconnected protein positioning it as a possible signalling hub. PPP2CA is the catalytic subunit of Protein Phosphatase 2 A (PP2A) which has been implicated in regulation of chondrogenesis^41^^,^^42^, chondrocyte cell cycle^43^ as well as in mediating anti-apoptotic effect of TGFβ1 on chondrocytes^44^. Besides PPP2CA, HSP90 was among the most interlinked proteins. Interestingly, HSP90 chaperone affects stability of BMAL1 and circadian gene expression^45^ and was proposed as a possible drug target in OA^46^, ^47^, ^48^.

In addition to intra-cellular proteins, a number of ECM proteins were shown to be rhythmic, suggesting that the circadian rhythm could influence cartilage tissue composition. Rhythmic changes in the abundance of adhesion molecules suggest that chondrocytes may change the way they interact with ECM during a 24-h cycle. We also found rhythmicity in a number of cytoskeleton associated proteins in cartilage, suggesting that the interaction between chondrocyte and its environment may also be time-of-day dependent. Indeed, the circadian clock in fibroblasts has been shown to modulate the efficiency of actin-dependent processes such as cell migration and adhesion, which ultimately impact the efficacy of wound healing^49^. Among the ECM proteins, we detected rhythmicity in the abundance of two lysyl hydroxylases PLOD1 and PLOD2 which could suggest a circadian rhythm in the maturation of collagen. However, we were not able to detect rhythmicity in mRNA or in protein abundance of collagen type II in cartilage neither when quantified as a whole protein (all peptides detected) nor in peptides belonging to the C-terminal propeptide of procollagen α1(II). Therefore, unlike collagen type I which is rhythmically secreted and assembled in the mouse tendon^21^, the synthesis of collagen type II does not seem to be rhythmic. It is still possible that the maturation of collagen type II is rhythmic as it has been shown that collagen in chick cartilage can be hydroxylated after the synthesis of protocollagen polypeptide^50^. Future studies are warranted to identify key physiological processes in mouse and human articular cartilage that show time-of-day dependent changes.

A caveat of the study is the possibility that the solubility of certain cartilage matrix proteins may differ between night and day as a result of altered mechanical loading. Changes in structure of ECM are known to occur following injury or as a result of osteoarthritis^51^ and may result in changes in solubility of cartilage proteins. In our previous work on mouse cartilage^8^ we observed age-dependent differences in solubility of proteins in sequential high salt (NaCl) and chaotropic (GuHCl) extracts. However, in the current study, we used the chaotropic extraction method which only involves denaturing and reducing conditions and allows solubilisation of majority of proteins that are not covalently bound to the insoluble portion of the ECM. As such, this harsh protein extraction method has minimised the influence of protein extractability.

The emerging importance of the circadian rhythm in physiology adds a new dimension to the complexity of biological systems and poses new challenges to understanding disease processes and biomarker discovery. Our analysis of published proteomic datasets revealed over 20% of rhythmic proteins to be dysregulated in osteoarthritis or with aging. Changes in the abundance of these proteins could be due to loss of circadian clock function or change of circadian phase. Indeed, we have previously shown that IL-1 dampens the clock rhythm in cartilage and that expression of clock components is dysregulated in osteoarthritis^5^^,^^52^. Therefore, caution should be exercised when measuring osteoarthritis biomarkers as some of them may be rhythmic in abundance and time of sampling could have a significant effect on results. Finally, understanding rhythmic processes in cartilage may allow optimization of existing osteoarthritis therapies, or the identification of new approaches for restoring tissue homeostasis, e.g., through resetting of the circadian clock.

## Contributors

Q-JM and MD designed the study and wrote the manuscript. MD, CA, JPDR performed the experiments. MD, CA, PW, VM and CL performed the bioinformatics analysis. MD, CA, JPDR, PW, VM, CL, JS, KEK, JAH, SRL, JFB and Q-JM analysed the data.

## Funding

This work was supported by an 10.13039/501100012041Versus Arthritis UK Senior Research Fellowship Award (20875, to Q-JM); an MRC project grant (MR/K019392/1, to Q-JM); a BBSRC David Phillips Fellowship (BB/L024551/1, to JS); a BBSRC sLoLa grant (BB/T001984/1 to KEK, JS and Q-JM); a National Health and Medical Research Council of Australia Project Grant GNT1063133 (to JFB) and Victorian Government's Operational Infrastructure Support Program (to JFB and SRL); a RUBICON secondment fellowship to MD (European Union funded project H2020-MSCA-RISE-2015_690850); and Wellcome Trust funding for The Wellcome Centre for Cell–Matrix Research (grant 203128/Z/16/Z).

## Conflicts of interest

No competing interests.

## Figures and Tables

**Supplementary Fig. 2:**