# Supplementary material for: Circadian time series proteomics reveals daily dynamics in cartilage physiology
Source: Osteoarthritis Cartilage. 2021 May;29(5):739–49. doi: 10.1016/j.joca.2021.02.008 (PMC8113022; doi:10.1016/j.joca.2021.02.008)
Supplement: Multimedia component 2 [file mmc2.docx]

| UniprotKB | Gene Name | Protein Name | Change | Condition | Ref |
| --- | --- | --- | --- | --- | --- |
| E9PV24 | FGA | Fibrinogen alpha chain | up | Intact vs. Degraded | [1] |
| O35074 | **PTGIS** | Prostacyclin synthase | up | Intact vs. Degraded | [1] |
| P22777 | **SERPINE1** | Plasminogen activator inhibitor 1 (PAI-1) | up | Intact vs. Degraded | [1] |
| Q9WVJ9 | EFEMP2 | Fibulin-4 | down | Intact vs. Degraded | [1] |
| Q61503 | **NT5E** | 5'-nucleotidase (5'-NT) | up | High grade OA vs. Low | [2] |
| P06801 | **ME1** | NADP-dependent malic enzyme | down | High grade OA vs. Low | [2] |
| Q9QYG0 | **NDRG2** | N-myc downstream-regulated gene 2 | down | High grade OA vs. Low | [2] |
| E9PV24 | FGA | Fibrinogen alpha chain | up | Normal vs. OA | [3] |
| P14824 | **ANXA6** | Annexin A6 | down | Normal vs. OA | [3] |
| P55264 | ADK | Adenosine kinase | down | Normal vs. OA | [4] |
| Q93092 | TALDO1 | Transaldolase | up | Normal vs. OA | [5] |
| P14824 | **ANXA6** | Annexin A6 | up | Normal vs. OA | [6] |
| Q8VDM4 | PSMD2 | 26S proteasome non-ATPase regulatory subunit 2 | up | Normal vs. OA | [6] |
| Q9JKF1 | IQGAP1 | Ras GTPase-activating-like protein | up | Normal vs. OA | [6] |
| Q9DBG3 | AP2B1 | Adaptor protein complex AP-2 subunit beta | up | Normal vs. OA | [6] |
| P47757 | CAPZB | F-actin-capping protein subunit beta (CapZ beta) | up | Normal vs. OA | [6] |
| P68254 | **YWHAQ** | 14-3-3 protein theta | up | Normal vs. OA | [6] |
| P08752 | GNAI2 | Guanine nucleotide-binding protein G(i) subunit alpha-2 | up | Normal vs. OA | [6] |
| Q8VDN2 | ATP1A1 | (Na(+)/K(+) ATPase alpha-1 subunit | up | Normal vs. OA | [6] |
| Q9DBF1 | ALDH7A1 | Alpha-aminoadipic semialdehyde dehydrogenase | up | Normal vs. OA | [6] |
| P07901 | HSP90AA1 | Heat shock protein HSP 90-alpha (Heat shock 86 kDa) | up | Normal vs. OA | [6] |
| P52480 | **PKM** | Pyruvate kinase PKM | down | Normal vs. OA | [7] |
| Q9D2G9 | HHIPL2 | HHIP-like protein 2 | up | Normal vs. OA | [7] |
| P51942 | MATN1 | Cartilage matrix protein (Matrilin-1) | down | Young vs. Old (mouse) | [8] |
| O88322 | NID2 | Nidogen-2 | down | Young vs. Old (mouse) | [8] |
| Q8BWT1 | **ACAA2** | 3-ketoacyl-CoA thiolase, mitochondrial | up | Young vs. Old (mouse) | [8] |
| Q91VD9 | NDUFS1 | NADH-ubiquinone oxidoreductase 75 kDa subunit | up | Young vs. Old (mouse) | [8] |
| P47199 | CRYZ | Quinone oxidoreductase | up | Young vs. Old (mouse) | [8] |
| Q9DBF1 | ALDH7A1 | Alpha-aminoadipic semialdehyde dehydrogenase | up | Young vs. Old (mouse) | [8] |
| Q8VDN2 | ATP1A1 | Na(+)/K(+) ATPase alpha-1 subunit | up | Young vs. Old (mouse) | [8] |
| P97807 | FH | Fumarate hydratase, mitochondrial (Fumarase) | up | Young vs. Old (mouse) | [8] |
| P17563 | SELENBP1 | Methanethiol oxidase (MTO) | up | Young vs. Old (mouse) | [8] |
| Q9CR68 | UQCRFS1 | Cytochrome b-c1 complex subunit Rieske, mitochondrial | up | Young vs. Old (mouse) | [8] |
| P26040 | EZR | Ezrin (Cytovillin) | up | Young vs. Old (mouse) | [8] |

**Supplementary Table 4. Analysis of published proteomic datasets comparing normal vs. osteoarthritic cartilage and young vs. aged cartilage.** Rhythmic proteins dysregulated in more than one study were highlighted in red. Genes rhythmic at both mRNA and protein level were shown in blue.

1. Steinberg J, Ritchie GRS, Roumeliotis TI, Jayasuriya RL, Clark MJ, Brooks RA, et al. Integrative epigenomics, transcriptomics and proteomics of patient chondrocytes reveal genes and pathways involved in osteoarthritis. Scientific reports. 2017;7(1):8935. Epub 2017/08/23. doi: 10.1038/s41598-017-09335-6. PubMed PMID: 28827734; PubMed Central PMCID: PMC5566454.

2. Steinberg J, Brooks RA, Southam L, Bhatnagar S, Roumeliotis TI, Hatzikotoulas K, et al. Widespread epigenomic, transcriptomic and proteomic differences between hip osteophytic and articular chondrocytes in osteoarthritis. Rheumatology. 2018;57(8):1481-9. Epub 2018/05/10. doi: 10.1093/rheumatology/key101. PubMed PMID: 29741735; PubMed Central PMCID: PMC6055583.

3. Wu J, Liu W, Bemis A, Wang E, Qiu Y, Morris EA, et al. Comparative proteomic characterization of articular cartilage tissue from normal donors and patients with osteoarthritis. Arthritis and rheumatism. 2007;56(11):3675-84. Epub 2007/10/31. doi: 10.1002/art.22876. PubMed PMID: 17968891.

4. Guo D, Tan W, Wang F, Lv Z, Hu J, Lv T, et al. Proteomic analysis of human articular cartilage: identification of differentially expressed proteins in knee osteoarthritis. Joint, bone, spine : revue du rhumatisme. 2008;75(4):439-44. Epub 2008/05/13. doi: 10.1016/j.jbspin.2007.12.003. PubMed PMID: 18468937.

5. Lambrecht S, Verbruggen G, Verdonk PC, Elewaut D, Deforce D. Differential proteome analysis of normal and osteoarthritic chondrocytes reveals distortion of vimentin network in osteoarthritis. Osteoarthritis and cartilage. 2008;16(2):163-73. Epub 2007/07/24. doi: 10.1016/j.joca.2007.06.005. PubMed PMID: 17643325.

6. Tsolis KC, Bei ES, Papathanasiou I, Kostopoulou F, Gkretsi V, Kalantzaki K, et al. Comparative proteomic analysis of hypertrophic chondrocytes in osteoarthritis. Clinical proteomics. 2015;12(1):12. Epub 2015/05/07. doi: 10.1186/s12014-015-9085-6. PubMed PMID: 25945082; PubMed Central PMCID: PMC4415313.

7. Ikeda D, Ageta H, Tsuchida K, Yamada H. iTRAQ-based proteomics reveals novel biomarkers of osteoarthritis. Biomarkers : biochemical indicators of exposure, response, and susceptibility to chemicals. 2013;18(7):565-72. Epub 2013/08/14. doi: 10.3109/1354750X.2013.810667. PubMed PMID: 23937207; PubMed Central PMCID: PMC3836424.

8. Li K, Zhang Y, Zhang Y, Jiang W, Shen J, Xu S, et al. Tyrosine kinase Fyn promotes osteoarthritis by activating the beta-catenin pathway. Annals of the rheumatic diseases. 2018;77(6):935-43. Epub 2018/03/21. doi: 10.1136/annrheumdis-2017-212658. PubMed PMID: 29555825.
